# Parental food-related behaviors and family meal frequencies: associations in Norwegian dyads of parents and preadolescent children

**DOI:** 10.1186/1471-2458-13-820

**Published:** 2013-09-10

**Authors:** Elisabeth L Melbye, Torvald Øgaard, Nina C Øverby, Håvard Hansen

**Affiliations:** 1 University of Stavanger, UiS Business School, Stavanger 4036, Norway; 2 University of Stavanger, Norwegian School of Hotel Management, Stavanger 4036, Norway; 3 University of Agder, Department of Public Health, Sport and Nutrition, PO Box 422, Kristiansand 4604, Norway

**Keywords:** Child eating, Family meals, Feeding practices, Home food environment

## Abstract

**Background:**

Frequent family meals are associated with healthy dietary behaviors and other desirable outcomes in children and adolescents. Therefore, increased knowledge about factors that may increase the occurrence of family meals is warranted. The present study has its focus on the home food environment, and aims to explore potential associations between parent-reported feeding behaviors and child-reported family meal frequencies.

**Methods:**

Cross-sectional surveys were performed among 10-12-year-olds and their parents recruited from eighteen schools in southwest Norway. The child questionnaire included measures of family meal frequencies (breakfast, dinner and supper). The parent questionnaire included measures of parental feeding behaviors adapted from the Comprehensive Feeding Practices Questionnaire. A series of multiple linear regression analyses were conducted to examine the relationships between parental feeding behaviors and the frequency of family meals.

**Results:**

The frequency of family breakfasts was associated with three parental feeding variables; *home environment* (β=.11, p<.05), *pressure to eat* (β=.11, p<.01), and *monitoring* (β=.10, p<.05). The frequency of family dinners and suppers was associated with one parental feeding variable; *home environment* (β=.11, p<.01 and β=.12, p<.01 for dinners and suppers respectively).

**Conclusions:**

The *home environment* variable was the most important correlate of child-reported family meal frequencies in this study. Although further research is needed, our findings support the evident influence of parents and the home food environment on child and adolescent eating behavior, which in the present study was measured as the frequency of shared family meals.

## Background

The family meal is considered a foundational activity that has the potential to serve vital functions such as the socialization of children [1]. Family meals involve activities such as food shopping, meal preparation, eating and conversation; and they provide an opportunity for parents to model healthful eating behaviors and to make healthy food available to their children [2]. Research conducted during the last decade has suggested that regular family meals are important for promoting healthy dietary behavior in school-aged children and adolescents [3]– [6]. Both cross-sectional and longitudinal research suggest that that family meals are associated with increased fruit and vegetable intake [5]– [7], lower levels of extreme weight control behaviors (i.e. unhealthy dieting behaviors such as following a very strict diet, using food substitutes, using laxatives and/or diuretics, or making oneself vomit) [8,9], and better psychosocial health [10]. There is also some evidence that family meals may be protective against obesity [11]– [14]. Furthermore, family meal frequency has been associated with increased discussion and knowledge of nutrition-related topics among family members [15].

Recent research suggests that factors within the home environment, such as parenting style [16] and family cohesion [17] are associated with family meal patterns. The concept of parenting style captures two important aspects of parenting: parental *demandingness* (also referred to as behavioural control) and parental *responsiveness* (referred to as parental warmth or supportiveness) [18]. According to Baumrind [19] there are four types of parenting style based on whether the parents are high or low on demandingness and responsiveness: *authoritarian parents* are high in demandingness and directiveness, but low in responsiveness (i.e. they expect their orders to be obeyed without explanation); *permissive parents* (also referred to as “indulgent” or “nondirective” parents) are more responsive then they are demanding (i.e. they allow considerable self-regulation and avoid confrontation with their child); *disengaged parents* (also referred to as “uninvolved” parents) are low in both responsiveness and demandingness (i.e. they are neglectful toward their child); *authoritative parents* are both demanding and responsive (i.e. they are assertive but also supportive). Regarding parenting style and family meals, both cross-sectional and longitudinal data have indicated a positive association between an authoritative parenting style (which is characterized by empathy and respect for the child on the one hand, and clear boundaries and expectations on the other hand) and the frequency of family meals [16].

Family cohesion has been defined as “the emotional bonding that family members have toward one another” [20]. Welsh et al. [17] found a positive association between perceived family cohesion and family meal frequency. However, as their study was cross-sectional, it is unknown whether family cohesion determined family meal frequency or family meal frequency determined family cohesion. According to Berge et al. [16], further research is needed to identify more factors within the home environment that may increase the occurrence of family meals for children and adolescents. This is the starting point for the present study.

Parents are central in creating the affective environment (i.e. the emotional climate) of the home through their general parenting style, and in organizing family meals [16]. Moreover, they influence their children’s eating behaviors through specific feeding practices [21]. According to Patrick & Nicklas [22], feeding practices represent the parents’ approach to maintain or modify children’s eating behaviors, and can be categorized into three different “feeding styles” corresponding with Baumrind’s [19] parenting styles: *authoritarian feeding* (i.e. strict controlling practices such as restricting certain foods and forcing the child to eat other foods), *permissive/neglectful feeding* (i.e. little or no structure and control, allowing the child to eat whatever s/he wants at whatever quantities s/he wants – also termed “nutritional neglect”), and *authoritative feeding* (i.e. a balance between the strictly controlling style and the permissive/unstructured style). Several studies have explored the associations between parental feeding practices, parental feeding styles and child food consumption and/or weight status [23]– [26]. However, to our knowledge, no previous studies have explored the relationships between *parental feeding practices* and *the frequency of shared family meals.* Both feeding practices and the arrangement of family meals are parts of what may be referred to as “the home food environment”, which is defined as a range of factors within the family environment that may be relevant in influencing child diet and weight outcomes [11]. Moreover, both feeding practices and the frequency of family meals are associated with child diet [3,21,23]. Drawing on Patrick & Nicklas’ [22] definition of feeding practices (i.e. the parents’ approach to maintain or modify children’s eating behaviors), and the fact that parents are central in organizing family meals [16], parents’ arrangement of regular family meals may in itself be considered a “feeding practice” as shared family meals certainly give the parents opportunities to modify their children’s eating behaviors. What is more, several feeding practices are shown to be interrelated [27]. Following from this, we argue that it is plausible that some feeding practices may be associated with the frequency of family meals. Presenting such associations would further support the evident influence of parents and the home food environment on child eating behavior. The Comprehensive Feeding Practices Questionnaire (CFPQ) [28] includes a broad range of feeding practices that may be relevant to child outcomes, and was applied to assess parental feeding behaviors in the present study.

Most previous research on parental feeding behaviors and child eating has focused on young children [27]. In the current study, we focus on children on the onset of adolescence (10-12-year-olds). Adolescence is the period from about the age of eleven to the late teen years, and represents a transitional stage from childhood to adulthood. It is characterized by the elaboration of identity, and it is a time of growing independence when individuals want to make their own decisions including *what* and *when* to eat [29,30]. This stage is typically a time of gradual shift from parental to peer influence [31]. However, the eagerness of adolescents to take over responsibility for food choice and meals is not necessarily matched with their ability to make healthy decisions. Adolescents have a reputation for unhealthy eating [32,33], and studies show an increased prevalence of irregular meal patterns among youth [34,35]. Furthermore, nutrition interventions directed towards this group of the population have had mixed success [36]. Therefore, the influence of parents should be assessed at all stages of this “hand-over-of-control” period to assist in the development of concurrent parent and child/adolescent nutrition intervention programs [37]. The rationale for focusing on 10-12-year-olds in the present study is that children this age are still highly influenced by parents [38]. Accordingly, it might be easier to implement intervention programs involving parents among individuals within this age range than among older adolescents.

Following from the preceding paragraphs, the aim of the present study was to build upon and extend the current literature on parent–child feeding interactions by investigating the relationships between a wide range of parent-reported feeding behaviors and child-reported frequencies of family breakfast, dinner and supper in a sample of preadolescent children (10-12-year-olds) and their parents.

## Methods

### Participants and procedures

Participants were recruited through primary schools in two neighboring municipalities in southwest Norway. All primary schools in these municipalities were asked to participate in the study, and 18 out of 25 schools (72%) agreed. In total, 1466 grade 5 and 6 students and one of their parents were invited. First, parents’ survey packages including information letters, consent forms and self-administered questionnaires were distributed to the children at school with instructions to bring them home to be completed by one of their parents (the parent included was chosen by self-selection according to involvement in home food issues). Next, after receiving written consent from the parents, child questionnaires were distributed and completed by the students at school. The study was approved by the Norwegian Social Sciences Data Services (NSD), which is the Privacy Ombudsman for all the Norwegian universities, university colleges and several hospitals and research institutes.

We received 963 completed parent questionnaires (66%). Response rate ranged from 44 to 93% among participating schools. Of the 963 parent respondents, 85% were mothers. The average age of the parents was 39.8 years, and 91% of the sample was of Norwegian or other Nordic origin. Out of 865 students having written consent from their parents to participate in the study, 796 (92%) completed the child questionnaire. Of the 796 child respondents, 51% were girls. Average age was 10.8 years (SD=0.6 years).

### Measures

Both parent and child draft questionnaires, which were largely based on items and scales from previous studies, were pretested before running the main survey. The drafts were tested through interviews with parents (n=6) and students (n=8) not included in the main survey to check if any questions, wordings or scales were perceived as difficult to understand, easy to misunderstand, vague or ambiguous, strange, “stupid” or irrelevant. Alternative wordings, scales or ways of asking questions were discussed with them to enhance the understanding and relevance of the questionnaire for the target groups (Norwegian 10-12-year-olds and their parents). Feedback from parents and students was registered in a form developed for this purpose, and we continued to recruit pretest participants for interviews until no new feedback was given. Based on results from the pretest, the draft questionnaires were slightly modified to fit our populations of interest.

#### Parent questionnaire

The parent questionnaire included a Norwegian version of the Comprehensive Feeding Practices Questionnaire (CFPQ) [27,28]. The CFPQ is a fairly new and not yet established instrument for measuring parental food-related behaviors. It is more comprehensive than previous feeding measures and includes 12 dimensions on parental feeding practices: *Child control* (parents allow the child control of his/her eating behavior and parent–child feeding interactions), *emotion regulation* (parents use food to regulate the child’s emotional status), *encourage balance and variety* (parents promote well-balanced food-intake, including the consumption of varied foods and healthy food choices), *home environment* (parents make (un)healthy foods available in the home), *food as reward* (parents use food as reward for child behavior), *involvement* (parents encourage child involvement in meal planning and preparation), *modeling* (parents actively demonstrate healthy eating for the child), *monitoring* (parents keep track of their child’s intake of less healthy foods), *pressure to eat* (parents pressure the child to consume more foods at meals), *restriction for health* (parents control the child’s food intake with the purpose of limiting less healthy foods and sweets), *restriction for weight control* (parents control the child’s food intake with the purpose of decreasing or maintaining the child’s weight), and *teaching about nutrition* (parents use explicit didactic techniques to encourage the consumption of healthy foods). A validation study by Melbye et al. [27] largely supports the validity of the CFPQ with parents of 10-12-year-olds in a Norwegian setting, thus we considered this instrument appropriate for assessing parental feeding behaviors in the present study.

Since previous studies have shown that parental socio-economic status (SES) may be an important correlate of both parent and child nutrition-related behaviors [39,40], we included the variables *parental educational level* and *household income* in our study to control for potential confounding effects of these factors.

#### Child questionnaire

The child questionnaire included three questions on family meal frequencies, serving as a measure of child eating behavior in the current study: 1) “How often do you eat breakfast together with your mother or your father?” 2) “How often do you eat dinner together with your mother or your father?”, and 3) “How often do you eat supper together with your mother or your father?” The meal frequency questions were adapted from the cross-European Pro Children study, where they were extensively tested and applied in several European countries including Norway [41]. Thus, we considered them valid for measuring family meal frequencies among 10-12-year-olds in the present study. All questions had 9 response categories (never=1, less than once a week=2, once a week=3, twice a week=4,…., six times a week=8, every day=9). The 9 response categories were re-coded to reflect meal frequencies in times per week for all variables prior to data analyses (never=0, less than once a week=0.5, once a week=1, twice a week=2,…, six times a week=6, every day=7). Thus, all response categories had a common denominator (times per week), which improved the readability of the results.

In Norway, breakfast is usually sandwiches or cereals, dinner is a hot meal, and supper often consists of sandwiches or cereals. Many Norwegians have supper, because they have an early dinner (at 16.00-18.00 hours) [42]. The lunch meal was not included in this study because an average Norwegian lunch normally consists of packed sandwiches eaten at school/work, and not together with the family.

### Data analyses

The SPSS statistical software package version 18 was used for all data analyses. First, Cronbach’s alphas were computed to measure the internal consistency of the CFPQ scales. Then CFPQ scale composites (average scores) were made, and means and standard deviations were calculated for both parent-reported (CFPQ-based) feeding variables and child-reported family meal frequencies. Next, bivariate correlation analyses were run between all variables to test for multicollinearity, and to get a first impression of relations between independent and dependent variables. We applied a cut-off value of 0.80 or greater for multicollinearity, as suggested by Haerens et al. [43].

To examine the associations between parent-reported feeding behaviors and child-reported family meal frequencies (which serves as a measure of child eating behavior in the present study), a series of multiple linear regression analyses were conducted with frequencies of family breakfast (model 1), dinner (model 2) and supper (model 3) as dependent variables. We chose a rather rigorous approach to our data, and listwise deletion was applied for all model analyses. Thus, only dyads with complete data sets for each of the three models tested were included in these analyses (regressions on family breakfast: n=630, regressions on family dinner: n=637, regressions on family supper: n=631). Independent samples t-tests were conducted to test for potential differences between dyads included in model analyses and those not included due to inadequate data.

## Results

Means, standard deviations and Cronbach’s alphas for parental feeding behaviors (CFPQ-based variables) are presented in Table 1. Means and standard deviations for family meal frequencies (breakfast, dinner and supper) are presented in Table 2. No multicollinearities were found between the independent (CFPQ-based) variables. Bivariate correlation analyses between independent and dependent variables indicated that several parent-reported feeding behaviors were related to child-reported family meal frequencies (see Table 3).

**Table 1:** Means (M), standard deviations (SD) and Cronbach’s alphas (α) for parental feeding practices (CFPQ-based variables)

**Variable/scale (number of items)**	**M**	**SD**	**α**
Monitoring (4)	4.05	0.56	.84
Child control (5)	2.38	0.58	.55
Encourage balance and variety (4)	4.47	0.51	.66
Home environment (4)	3.92	0.68	.57
Involvement (3)	3.46	0.83	.67
Pressure to eat (3)	2.77	0.97	.61
Restriction for weight (8)	2.20	0.80	.83
Food as reward (2)	1.56	0.79	.69
Restriction for health (4)	2.88	1.00	.73
Teaching nutrition (3)	4.13	0.66	.44
Modeling (4)	3.86	0.74	.66
Emotion regulation (1)	1.47	0.75	-

**Table 2:** Means (M) and standard deviations (SD) for family meal frequencies (times per week)

**Meal**	**M**	**SD**
Breakfast	4.02	2.64
Dinner	6.63	1.08
Supper/evening meal	3.30	2.81

**Table 3:** Pearson’s correlations between parental feeding practices (independent variables) and family meal frequencies (dependent variables)

**Parental feeding practices**	**Family breakfast frequency**	**Family dinner frequency**	**Family supper frequency**
Monitoring	.11*	.04	.02
Child control	-.09**	-.11*	-.03
Encourage balance and variety	.06	.08**	.04
Environment	.13*	.09**	.12*
Involvement	.01	-.00	.02
Pressure to eat	.08**	.00	.01
Restriction for weight	-.01	-.04	-.02
Food as reward	-.02	-.09**	-.00
Restriction for health	-.05	-.06	-.05
Teaching nutrition	.10*	.06	.09**
Modeling	.11*	.06	.08**
Emotion regulation	-.02	-.08**	.01

* p<.01, ** p<.05.

Regressions on child-reported family breakfast frequency (model 1) revealed that three parental feeding practices were significantly and positively related to the frequency of family breakfasts: *Home environment* (β=.11, p<.05), *pressure to eat* (β=.11, p<.01), and *monitoring* (β=.10, p<.05). Only the *home environment* variable was significantly (and positively) related to the frequency of family dinners (model 2; β=.11, p<.01) and suppers (model 3; β=.12, p<.01) respectively (see Table 4). These findings are discussed in more detail in the discussion section.

**Table 4:** Results from multiple regression analyses predicting family meal frequencies from parental feeding practices (CFPQ-based variables)

**Parental feeding practices**	**Family breakfast frequency(Model 1)**	**Family dinner frequency(Model 2)**	**Family supper frequency(Model 3)**
Monitoring	.10***	.02	-.03
Child control	-.03	-.08	.00
Encourage balance and variety	-.02	.02	-.03
Environment	.11**	.11**	.12**
Involvement	-.01	-.02	-.01
Pressure to eat	.11**	.03	.01
Restriction for weight	.02	-.03	-.01
Food as reward	.02	-.07	.00
Restriction for health	-.06	-.02	-.04
Teaching nutrition	.04	-.05	.06
Modeling	.05	.03	.05
Emotion regulation	.01	-.01	.05
R^2^	.08*	.04 (n.s.)	.03 (n.s.)

* p<.001, ** p<.01, *** p<.05.

NOTE: Potential confounding by parental SES (i.e. parental educational level and household income) was adjusted for in all model analyses.

As mentioned in the methods section, independent-samples t-tests were conducted to compare variable scores (model variables and socio-demographic variables) for parent–child dyads included in model analyses and those not included due to incomplete data. Of the more than twenty variables tested, we found only one variable with significantly different scores for dyads included and dyads not included: Parent-reported child control (M=2.41, SD=0.57 for dyads included and M=2.29, SD=0.59 for dyads not included, t(725)=1.93, p=0.05). The magnitude of the difference in means was very small (mean difference=0.12, eta squared=0.005), suggesting that the differences between dyads included and dyads not included in our model analyses were negligible.

## Discussion

The aim of the present study was to investigate the relationships between parent-reported feeding behaviors and child-reported family meal frequencies (breakfast, dinner and supper) in families of preadolescent children (10-12-year-olds). A series of multiple linear regressions revealed that some parental feeding behaviors were significantly associated with the frequency of family meals.

Starting with breakfast, we found that three parental feeding variables; *home environment*, *pressure to eat* and *monitoring* were positively associated with child-reported family breakfast frequency. Breakfast is widely perceived as the most important meal of the day [44,45]. Thus, the positive relation between the *home environment* variable and family breakfast frequency is reasonable, as parents with high scores on this variable (i.e. parents providing a healthy home food environment) will perhaps be more inclined to see the importance of the breakfast meal and share it with their children. A high frequency of family breakfasts will also give parents more opportunities to actively control the amount of food eaten by their child for breakfast, thus making sure that he or she is sufficiently nourished before s/he goes to school. Therefore, the positive relation between the parental *pressure to eat* variable and family breakfast frequency also seems rational. For similar reasons, the positive relation between parental *monitoring* and family breakfast frequency seems logical, as an increased frequency of family breakfast gives increased possibilities to keep track of what the child eats (at least for breakfast).

Regarding dinner and supper, only the *home environment* variable was (positively) associated with the frequency of shared meals. As for breakfast, the positive relations between the *home environment* variable and family dinner and supper frequencies are reasonable, as parents with high scores on this variable (i.e. parents providing a healthy home food environment) will perhaps be more inclined to see the importance of sharing meals with their children. Thus, one possible mechanism of the associations found may be that providing a healthy home food environment promotes the occurrence of family meals. However, it may also be the opposite; that the occurrence of frequent family meals is simply a marker of a healthy home food environment. According to Berge et al. [16] the organizing, preparing and eating of a family meal may be a stress-inducing event. Therefore, one may speculate that families where parents provide a healthy home food environment may be more organized and structured when it comes to food and eating, thus being more able to successfully arrange frequent family meals.

Concerning the means (M) and standard deviations (SD) of the meal frequencies in our sample (Table 2), it is worth noting that the mean family dinner frequency (M=6.63) was considerably higher and its standard deviation (SD=1.08) considerably lower than the corresponding numbers for shared breakfasts (M=4.02, SD=2.64) and suppers (M=3.30, SD=2.81). This finding illustrates the generally high frequency and low variance of shared family dinners, as most Norwegian children this age share the dinner meal with their parents [46]. The frequency of family dinners has been associated with adolescents’ healthy eating, but also with other health- and well-being related assets such as increased positive self-identity and reduced risk-behaviors [47]. Thus, the family dinner may function as an important habit forming institution with a widespread effect on children’s and adolescents’ general health and well-being. Accordingly, the finding of a high frequency (and low variance) of family dinners in our sample is a favorable one.

As articulated in the background section, the very definition of feeding practices (i.e. the parents’ approach to maintain or modify children’s eating behaviors), and the fact that parents are central in organizing family meals [16], suggest that parental arrangement of regular family meals may be considered a feeding practice in itself (i.e. the family meal gives the parents opportunities to modify their children’s eating behaviors). Furthermore, several feeding practices are shown to be interrelated [27]. Following from this, we argued that some feeding practices may be associated with child-reported frequencies of family meals. Thus, our results revealing positive associations between some parental feeding practices and child-reported frequencies of family breakfast, dinner and supper support the notion that arranging regular family meals may be considered a feeding practice in itself. Moreover, the feeding practices found to be associated with child-reported family meal frequencies are considered controlling feeding behaviors, which again suggests that parents with a regulatory approach to their children’s eating behavior have children who share more meals with their parents. Some studies have reported associations between strict controlling feeding practices (e.g. heavy restrictions on unhealthy foods, heavy pressure to eat healthy foods, and orders to “clean the plate”) and undesirable child outcomes such as lower intakes of fruit, juice and vegetables [48]– [50], fixation and over-consumption of high-fat and high-sugar foods [51], lower sensitivity to psychological cues of satiety [52], and excessive weight gain [51,53]. However, other studies have shown associations between controlling feeding practices and healthful child outcomes such as increased consumption of fruit and vegetables and decreased consumption of snack foods [54,55], thus indicating that some controlling feeding practices may actually be beneficial, guiding children to eat in a more healthful manner. What may be logically deduced from the preceding discussion is that a regulatory approach, including certain controlling feeding practices and the arrangement of frequent family meals, could be a favorable parental strategy to influence child eating behaviors.

Among the strengths of this study is that it has reports from two different sources; parents and children. Thus, the “common methods problem” is reduced compared to situations where only one data source is available (e.g. parents reporting both feeding behaviors and family meal frequencies). Another strength is its large sample size, which increases the validity of the results. A few limitations also need to be commented: Although the response rate in the present study was quite high (72% for schools, 66% for parents and 92% for children having written consent from their parents), potential non-response bias cannot be ruled out. However, non-response at the school level is not considered problematic here, as the schools that chose to decline explained their nonparticipation as a result of substantial involvement in another school based project (i.e. a project intended to prevent problem behavior such as bullying) and that participation in an additional project would be too time consuming. What is more, the invited schools were all located within the same geographic area, which according to Statistics Norway (SSB) is an area with high and still growing labor attachment and household incomes. Therefore, and because the Norwegian school system is based on a socio-democratic principle of equality, we consider the chances of non-response bias caused by differences in response rates between “high-SES” and “low-SES” schools negligible. At the parent/student level, on the other hand, there is a chance that some groups may be under-represented in our study sample. Several health-related studies have shown that response-rates vary according to socio-demographic variables, with lower response rates in individuals from lower socio-economic classes and among non-western immigrants [56]– [58]. This may also be the case in the present study. Thus, if invitees from lower socio-economic classes and non-western immigrant groups systematically excluded themselves from our survey, the results obtained may not be fully representative for the population of interest (here: dyads of parents and their 10-12-year old children). Therefore, the results from this study should be interpreted with caution, as should all studies based on self-selection. That said; as the attendance in health-related studies tend to vary according to socio-demographic variables [57], and since Norway is a rather homogeneous country [59], we believe our results are likely to be generalizable to other areas in Norway with a social distribution among residents that is similar to the one in the present study sample.

Another obvious limitation is the cross-sectional design, which makes us unable to confirm the direction of the associations found. Because parents’ arrangement of family meals may be considered a feeding practice in itself, our finding of associations between some feeding practices measured by the CFPQ and child-reported frequencies of family meals seems rational. However, as suggested above, there may be different potential mechanisms explaining these associations. For example, providing a healthy home food environment and using controlling feeding practices such as pressure to eat and monitoring may promote the occurrence of family meals, or the other way around; the occurrence of family meals may be a marker of a healthy home food environment and parental use of controlling feeding practices such as pressure to eat and monitoring. Nevertheless, according to recent literature, the parent–child feeding relationship is probably a dynamic and bi-directional one [60]– [62]; it includes different food related behaviors and characteristics in both parents (e.g. feeding practices, parenting style) and children (e.g. eating behaviors, weight status), and it changes over time and situations. Our findings make a contribution to the literature by unraveling important, yet underexplored associations within the child feeding domain, namely the relationships between parental feeding behaviors and the frequency of shared family meals. However, future research could benefit from applying designs more suitable to test for causality, and samples covering larger parts of the population. As parental arrangement of family meals may be considered a feeding practice in itself, we also suggest that the frequency of family meals is incorporated in future feeding practices measures, thus creating even more coherent and broad instruments. The development of more comprehensive instruments for measuring food-related variables within the home environment is warranted to provide a more complete picture of the parent–child feeding relationship. We strongly believe that increased knowledge of the associations between different factors within the home food environment is essential for developing effective nutrition interventions including both children/adolescents and their parents.

## Conclusions

A unique contribution of this study is its assessment of the relationships between parental feeding behaviors and family meal frequencies. Our results show that parent-reported feeding practices such as *home environment*, *pressure to eat* and *monitoring* are associated with child-reported frequencies of family meals. Although further research is needed, our findings support the evident influence of parents and the home food environment on child and adolescent eating behavior (here measured as child-reported frequencies of shared family meals). What is more, parents’ arrangement of family meals may be considered a feeding practice in itself and should therefore be incorporated in future feeding practices measures.

## Competing interests

The authors declare that they have no competing interests.

## Authors’ contributions

ELM designed the study, collected and analyzed the data, and drafted the manuscript. TØ, NCØ and HH contributed to the writing of the article. All authors read and approved the final manuscript.

## Pre-publication history

The pre-publication history for this paper can be accessed here:

http://www.biomedcentral.com/1471-2458/13/820/prepub
